# Applied Anatomy of Perimembranous Ventricular Septal Defect for Transcatheter Device Closure

**DOI:** 10.1016/j.jacasi.2026.03.002

**Published:** 2026-07-07

**Authors:** Thao V.N. Nguyen, Siew Yen Ho, Tin N. Do

**Affiliations:** a Cardiology Department, Children’s Hospital 1, Ho Chi Minh City, Vietnam; b Department of Pediatric Cardiology, Royal Brompton Hospital, Guy's and St Thomas' NHS Foundation Trust, London, UK; c National Heart and Lung Institute, Imperial College London, UK; d Interventional Cardiology Department, Children’s Hospital 1, Ho Chi Minh City, Vietnam; e University of Medicine and Pharmacy, Ho Chi Minh City, Vietnam

**Keywords:** cardiac morphology, perimembranous ventricular septal defect, transcatheter device closure

## Abstract

Perimembranous ventricular septal defect is a common congenital heart disease effectively treated by transcatheter device closure with high success rates and minimal complications. This review categorizes perimembranous ventricular septal defects into 7 morphologic variants based on anatomical extension and correlates these with echocardiographic classifications: type 1 is central perimembranous; type 2 is superior-anterior extension; type 3 is superior-posterior extension; type 4 is inferior-anterior extension; type 5 is inferior-posterior extension; type 6 is confluent extensions with more than one extension direction; and type 7 is complex type with outlet septum involvement. Type 2 defects are often associated with aortic valve prolapse and regurgitation, requiring soft, flexible devices to minimize valve injury. Type 5 defects lie close to the conduction system; deploying devices within an aneurysmal pouch helps prevent heart block and tricuspid regurgitation. Type 6 and 7 defects sometimes necessitate multiple devices for complete closure. Understanding these anatomical variants aids in selecting appropriate devices and optimizing procedural outcomes.

## Highlights

• The morphology of perimembranous ventricular septal defect (VSD) is highly complex due to its proximity to critical surrounding structures, particularly the aortic valve, tricuspid valve, and atrioventricular conduction system.
• Newly developed devices with advanced and anatomically specific designs show promise for safety and efficacy of perimembranous VSD closure.
• A detailed understanding of perimembranous VSD anatomy is essential for optimal device selection and achieving successful outcome.

Perimembranous ventricular septal defect (pmVSD) is one of the most common congenital heart defects with extremely good outcomes from surgery.^1^^,^^2^ In the evolution of transcatheter interventions with many new techniques and new generation innovative devices, many centers have performed ventricular septal defect (VSD) device closure and demonstrated promising outcomes with high success rates and minimal complications, especially an extremely low (1% to 5%) incidence of heart block.^3^, ^4^, ^5^, ^6^, ^7^, ^8^, ^9^

Surgical closure of VSDs typically involves placing a patch along the right ventricular (RV) side bordering the defect. Most of the morphologic descriptions of VSD, therefore, are based primarily on the RV aspect, without mention of the left ventricular (LV) aspect. However, in the context of transcatheter intervention, interventionalists must consider not only the size of the VSD but its margin from both RV and LV sides of the ventricular septum, including any structures in the immediate vicinity such as valvar and other septal structures and the atrioventricular conduction system. Furthermore, the ventricular septum being nonplanar can affect the spatial relationships of structures surrounding the VSD.

This broader anatomical perspective is critical particularly for pmVSD to avoid complications during device deployment and to ensure the success of the procedure. A pmVSD is defined as a defect with a fibrous posteroinferior border. In contrast to the completely muscular border found in a muscular VSD, the fibrous border signifies proximity to the anticipated site of the atrioventricular conduction bundle. In this paper, we begin by describing the anatomy of the normal ventricular septum and the atrioventricular conduction system, followed by a review of the morphologic variations of isolated pmVSD as seen on heart specimens and clinical imaging and, based upon the vast Asian experience of one of the authors (Dr Tin N. Do), some of the types of devices that may be deployed when considering transcatheter intervention.

## Anatomy of ventricular septum in the normal heart

**Figure 1:**
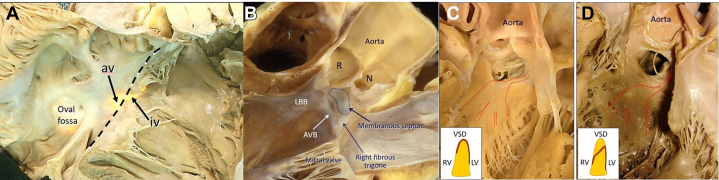
Membranous Septum Anatomy and Course of the Conduction Pathways (A, B) Normal hearts. (A) Membranous septum viewed from right ventricular (RV) side. Transillumination reveals the atrioventricular (av) part and interventricular (iv) parts of membranous septum divided by the hingeline (dashes) of the tricuspid valve. (B) Left ventricular (LV) view shows membranous septum (within dotted line). The atrioventricular bundle (AVB) and left bundle branch (LBB) covered by white tissue of the fibrous sheaths can be traced “off-crest,” away from the membranous septum. (C, D) LV views of hearts with perimembranous ventricular septal defect (pmVSD) to show that the AV conduction bundles (red lines) in the posteroinferior border of the pmVSD may be “on-crest” or “off-crest” position. N = Non-coronary cusp; R = right coronary cusp; VSD = ventricular septal defect.

For the most part, the cardiac septum is muscular apart from a very small area comprising of a thin sheet of fibrous tissue termed the membranous septum. Because of the hingeline (annulus) of the tricuspid valve’s septal leaflet transecting the membranous septum, together with the different levels of septal attachment of the mitral and tricuspid valvar hingelines, the location of the membranous septum thereby separates the LV from both the right atrium (RA) and the RV (Figure 1), although its RA component may be tiny.^10^ On the LV side, it directly borders the interleaflet triangle between the hingelines of right coronary cusp (RCC) and noncoronary cusp (NCC) of the aortic valve where it joins the right fibrous trigone as part of the central fibrous body (Figure 1*)*. Viewed from the RV side, the membranous portion represents the juncture of the 3 anatomical regions of the RV: the inlet, trabecular, and outlet.

At the site of the membranous septum the septal leaflet of the tricuspid valve (TV) frequently diminishes, partly covering the interventricular portion of the membranous septum in most hearts.^11^ Viewed from the RV, this fibrous septum’s anterosuperior border lies behind the septal insertion of the supraventricular crest which is an infolding on the ventricular wall continuing into the infundibulum supporting the pulmonary valve (PV). Notably, this area is not septal because the aortic valve lies immediately behind it. At the septum, the crest (also termed the ventriculo-infundibular fold) sits between the 2 limbs of the Y-shaped septomarginal trabeculation, a prominent muscle bundle of the septum.^12^ Normally, any outlet septum is indistinguishable from the crest.

For describing the location of VSDs, the muscular septum is considered in terms of 3 portions (inlet, trabecular, and outlet region), but there are no robust anatomical landmarks for each. The largest is the trabecular portion spanning between the inlet and outlet portions and from the membranous septum to the apex of the heart. The inlet portion lies inferior and posterior to the membranous septum, beginning at the level of the atrioventricular valves and extending apically to the level of their papillary muscle attachments. The outlet portion extends from the supraventricular crest and upper region of the septomarginal trabeculation to the PV. This portion is mainly the cone-shaped infundibulum above the septum that supports the PV.

In hearts with pmVSD, however, the outlet septum is distinct in the RV of many hearts and attached to the location of one of the limbs of the septomarginal trabeculation.

## Location of atrioventricular conduction system in pmVSD

In hearts with pmVSD, the fibrous posteroinferior border of the VSD is the area of fibrous continuity between the tricuspid and mitral valves or tricuspid, mitral, and aortic valves. It can bear a vestige of the membranous septum to varying extents. Where a sizeable membranous flap remains, it suggests that the conduction bundle is not immediately bordering the hole. Some surgeons suture the patch to it without risk to the conduction bundle.^13^ In those without any membranous remnant, continuity between tricuspid and mitral valves without interposition of muscle marks the fibrous border. In hearts with isolated pmVSDs, the atrioventricular node is normally located at the apex of the triangle of Koch. The penetrating bundle of His passes through the central fibrous body to the border of the membranous septum with the muscular ventricular septum. From there, the atrioventricular bundle may course directly upon the crest of the septum that is the margin of the VSD. In others, instead of coursing directly onto the immediate margin, the bundle may course slightly off the crest to the LV side before branching into the right and left bundle branches with the latter descending along the subendocardium (Figure 1), whereas the right bundle branch traverses through the muscular septum before emerging in the subendocardium at the base of the medial (Lancisi) muscle.^13^, ^14^, ^15^, ^16^ Less commonly, the bundle runs on the right side of the septal crest.^17^

## Applied anatomy of pmVSD

The echocardiographic classification of pmVSD proposed by Singhi et al^18^ for guiding transcatheter device closure is based on anatomic characteristics of the VSD with its intrinsic relation to the anteroseptal tricuspid commissure and the aortic annulus when visualized on echocardiography is highly applicable in pmVSD device closure.^18^ According to this classification, pmVSD can be divided into 4 main types. Type A is a VSD with an absent aortic rim. The superior margin of the defect flushes with the aortic annulus with no significant separation. Type B is a VSD with good well-formed aortic margin formed by the ventriculo-infundibular fold. Type C is a VSD that is restricted by septal aneurysm formed by fibrous tissue ingrowth from the edges of the VSD. Type D is a VSD that is restricted by septal aneurysm formed by part of TV leaflets, either septal or anterior, caused by the chordal attachments to the apical edge of the VSD. In this classification, the superior margin of the VSD is always separated from the aortic annulus in types B, C, and D.^18^ However, this echocardiographic classification focuses on the proximity of each border of the VSD rather than showing the VSD comprehensively in 3-dimensional anatomy. In fact, one defect can be classified into more than one category. For example, in both types A and C, the defect is close to the aortic valve in its superoanterior border but has inlet extension and aneurysmal tissue in its posteroinferior border.

**Figure 2:**
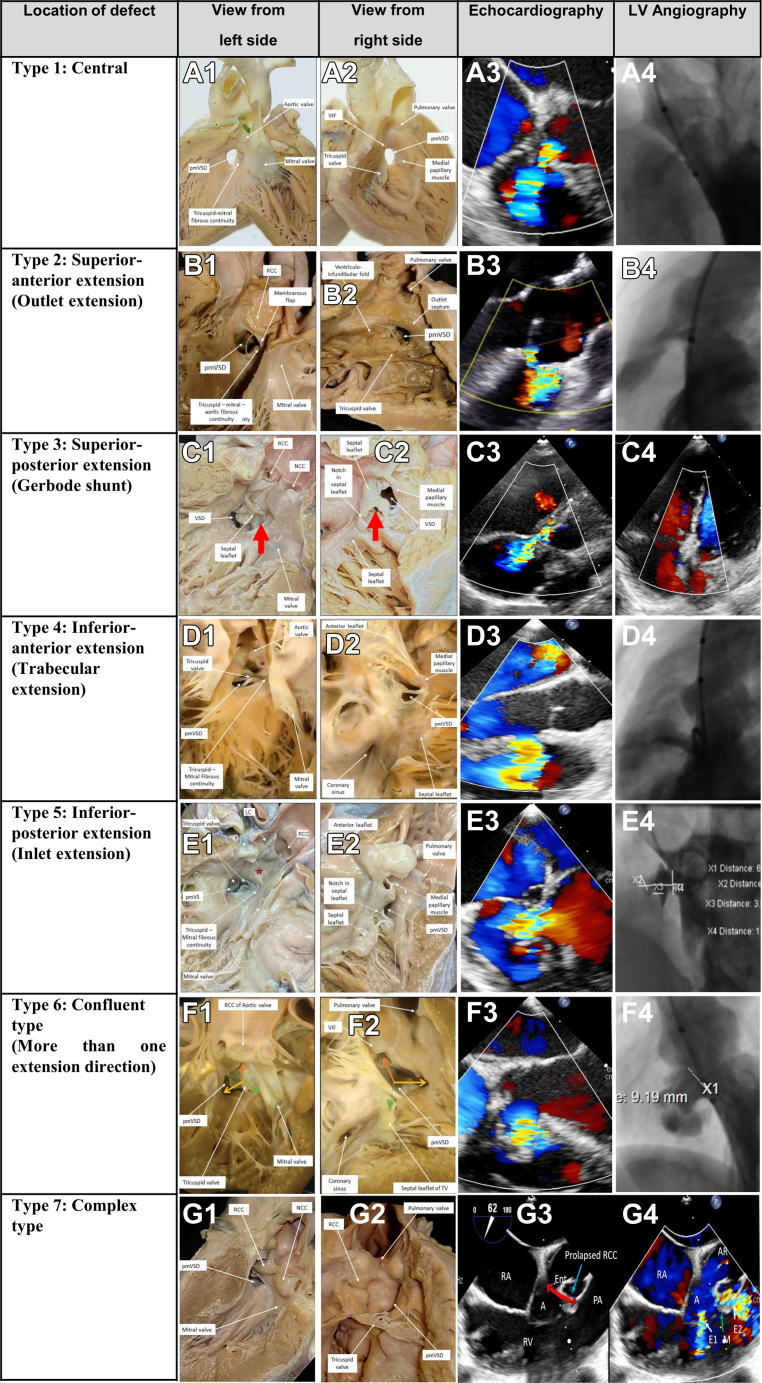
Morphology of pmVSD and Variants Depending on Its Extensions Type 1 includes central perimembranous defect, minimal extension. Type 2 includes outlet pmVSD with antero-superior extension. Type 3 includes pmVSD with posterosuperior extension, causing Gerbode shunt. Type 4 includes trabecular pmVSD with antero-inferior extension. Type 5 includes inlet pmVSD with poster-inferior extension, underlying the septal leaflet of the tricuspid valve. Type 6 includes confluent type pmVSD with 3 directions of extension where an orange arrow indicates antero-superior extension–outlet extension, a blue arrow indicates antero-inferior extension–trabecular extension, and a green arrow indicates posteroinferior extension–inlet extension. Type 7 is a complex type comprising membranous septum defect and outlet septum defect. Red stars (in A1, D1, E1) indicate muscular rim between the defect and the aortic valve. In outlet pmVSD (B1), a thin membranous flap (membranous remnant) is suspended between the superior defect border and the aortic valve. Red arrows (in C1, C2) indicate the sites of the Gerbode shunt owing to cleft in the septal leaflet. In complex type (G3, G4), a transesophageal echocardiography modified short-axis view shows the entrance extending beneath the noncoronary cusp (NCC) and the right coronary cusp (RCC), with the RCC prolapse partially covering the defect (blue arrow). On the RV side, the defect divides into 2 exit points (E1/E2): E1 located posteroinferiorly associated with aneurysm (A), and E2 located antero-superiorly just beneath the pulmonary valve and the pulmonary artery (PA). A muscular septal insertion in G4 (M, green arrow) is visible between the 2 exit points. AR = aortic regurgitation; Ent = ; LC = ; M = ; pmVS = ; TV = tricuspid valve; RA = right atrium; VIF = ventricular infundibular fold; other abbreviations as in Figure 1.

Anatomically, when a pmVSD affects only the membranous septum, it would be a very small defect. Most commonly, perimembranous defects are larger than the normal interventricular membranous septum due to extensions of the defect into muscular septum (Figure 2). Based on the defect’s location and considering its extensions, if any, toward 1 of the 3 regions of the ventricle, the defect is described accordingly: central pmVSD (minimal extension); outlet pmVSD (with outlet extension); inlet pmVSD (with inlet extension); trabecular pmVSD (with trabecular extension); and confluent pmVSD (when extensions are toward more than 1 of 3 regions).^19^^,^^20^ TV tissue and accessory tissue cover over some parts of the pmVSD in the last 3 variants.

In planning device closure, features such as proximity of the conduction bundles to the VSD margin, the fibrous border that includes a vestigial membranous septum, excessive TV tissue or aneurysmal pouch are relevant. Furthermore, a 3-dimensional perspective of the LV and RV aspects are needed. For example, there may be a minor malalignment of septal structures. In those with outlet extension, the presence of a muscular outlet septum with/without small degree of malalignment into the RV or LV outlet may provide a muscular aortic rim. In those with inlet extension and malalignment between the ventricular and atrial septums, TV tissue may traverse through the VSD.

Even in patients with isolated pmVSDs, because of the tremendous variability in morphology and bordering structures that may be encountered when planning transcatheter interventional closure, we propose a classification of pmVSD types (Central Illustration) correlating images from current imaging modalities with morphologic images and comment on features relevant to intervention and types of devices that may be appropriate. For convenience, the pmVSDs are listed morphologically as types 1 to 7 (Figure 2) with descriptions highlighting similarities and differences with the echocardiographic classification of pmVSD proposed types A to D by Singhi et al.^18^

**Central Illustration:**
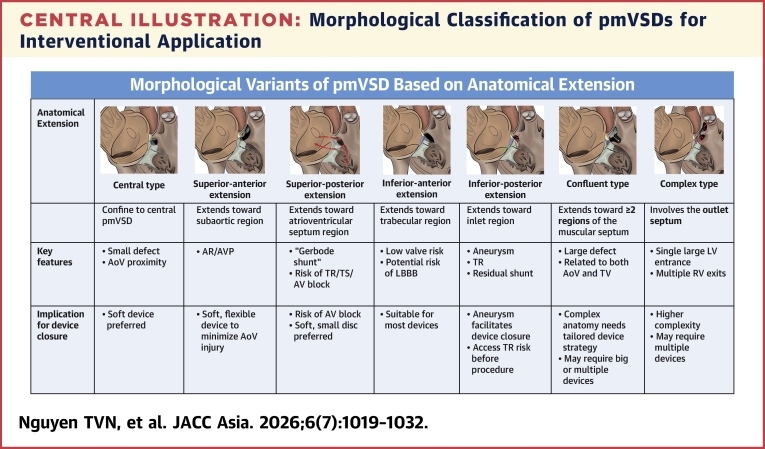
Morphological Classification of pmVSDs for Interventional Application Proposed seven morphological types of perimembranous ventricular septal defects (pmVSDs) based on their extensions. A = aneurysm; AR = aortic regurgitation; E1 = exit 1; M = ; RA = right arterial.

### Type 1: perimembranous central VSD

Defects occupying the location of the missing interventricular membranous septum are small. Termed central defects, they have minimal extensions into the muscular septum. Their fibrous border is the fibrous continuity between tricuspid, mitral, and aortic valves with or without a remnant of the membranous septum. This configuration results in a short, thin membranous aortic rim, which is typically insufficient to be considered a stable anchoring site for device closure. This type falls into echocardiographic type A due to the absence of muscular aortic rim, but the defect is usually small. Because of the close proximity to the aortic valve, trivial or mild aortic regurgitation — often detected only at a subclinical level — occurs more frequently in this anatomical subtype and must be closed earlier. Given this morphology, soft and small profile devices (Amplatzer duct occluder II [ADO II], Nit-Occlud Lê VSD, and LifeTech multifunctional occluder [MFO]) are preferred. An antegrade approach via the venous route with creation of an arteriovenous loop should be considered to allow precise control of LV disc deployment and to minimize interference with the aortic valve. In 20% of pmVSDs, fibrous continuity between the aortic and TV leaflets is absent,^19^ resulting in a muscular rim forming along the superior border of the defect, creating a separation between the VSD and the aortic valve above (Figure 2, red stars), and providing a stable superior rim to employ the device. This morphology resembles the echocardiographic type B of pmVSD. Because of the presence of a superior muscular rim, various conventional devices may be suitable for closure of this subtype; however, devices with a soft structure and low-profile design are preferred.

### Type 2: pmVSDs with outlet extension

**Figure 3:**
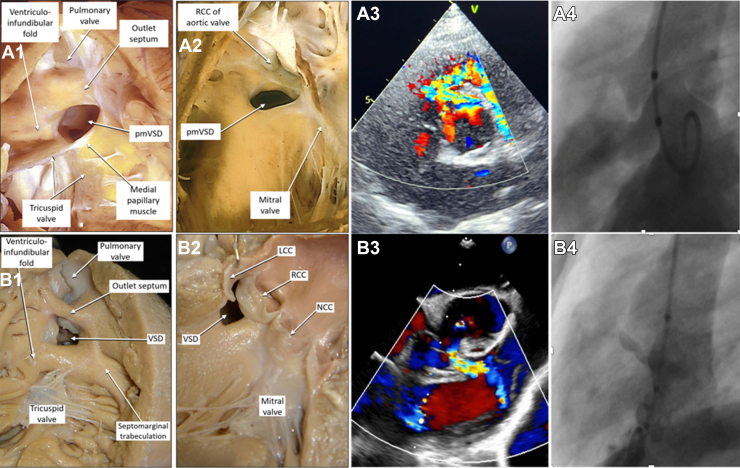
Comparison of Outlet pmVSD and Isolated Subaortic (Muscular Outlet) VSD (A1-A4) Outlet pmVSD. (A1, A2) pmVSD with outlet extension located below the outlet septum, maintaining separation from the pulmonary valve. (A3) Transthoracic echocardiogram. Parasternal short-axis view showing a large outlet pmVSD extending from TV to more than half of the RCC of the aortic valve; (A4) LV angiography showing large pmVSD extending to subaortic with mild prolapse of RCC. The shunt goes down to the RV and away from the pulmonary valve. (B1-B4) Isolated subaortic VSD. (B1, B2) Subaortic (muscular outlet) VSD with RCC prolapse. The VIF distances the defect from the tricuspid valve. (B3) On transesophageal echocardiogram, an isolated subaortic VSD is shown without perimembranous extension. The defect is located away from the septal leaflet, and the shunt jet is narrow and directed strictly anteriorly, pointing toward the right ventricular outflow tract (RVOT). (B4) LV angiography shows that the shunt is beneath the RCC and moves forward directly to the RVOT. LCC = left coronary cusp; other abbreviations as in Figures 1 and 2.

These are characterized by defects that extend anteriorly and cephalad toward the outlet septum.^20^ On the RV aspect, the defect extends in a cranial direction, often reaching up to or lying just beneath the supraventricular crest, which forms the boundary between the RV inflow and outflow tracts. On the LV side, the extension is beyond the interleaflet triangle situated between the RCC and the NCC of the aortic valve. The defect may continue superiorly and anteriorly toward the most anterior portion of the RCC (Figure 3A3). However, it typically does not involve the entire RCC hingeline.

One of the most clinically significant aspects of this anatomical subtype is its frequent association with aortic valve prolapse (AVP). Approximately 30% of pmVSDs with outlet extension show AVP, most commonly involving RCC and/or NCC.^21^ The prolapse results from the herniation of unsupported aortic leaflet into the VSD. This phenomenon can lead to aortic regurgitation (AR), which is mild to moderate in most cases, although severe AR has been reported in approximately 13% of affected patients.^21^ AVP and its resulting hemodynamic consequences carry important implications for procedural planning as they may influence both the timing of intervention and the selection of closure technique or device.

From an echocardiographic perspective, these lesions share features with type A pmVSDs.^18^ However, they differ significantly from small, centrally located pmVSDs, particularly in their larger size, closer proximity to the RCC, and higher incidence of AVP, all of which contribute to an increased risk of aortic valve impingement or post-procedural AR if device selection and positioning are suboptimal. The use of soft and flexible devices (eg, ADO II, Nit-Occlud Lê VSD, and MFO) may reduce the risk of aortic valve injury; however, these devices are generally larger in size compared with those used for type 1 pmVSDs.

This anatomical type must also be carefully distinguished from the isolated subaortic VSD (also referred to as a muscular outlet VSD that has completely muscular borders from the RV aspect), but it flushes with RCC of the aortic valve from the LV aspect (Figures 3B1 and 3B2). Those with completely muscular borders viewing from the RV aspect, including a narrow muscular rim separating the pulmonary and aortic valves and a muscular posterior rim separating the VSD and TV, have also been termed “intracristal VSD” (Figure 3B1) and are typically small and located beneath the most anterior portion of the RCC of the aortic valve.^22^ Unlike the broader jet seen in outlet pmVSDs, the left-to-right shunt jet in subaortic VSDs is characteristically narrow and directed strictly anteriorly toward the anterior wall of the right ventricular outflow tract (RVOT) (Figure 3). Because of the limited spatial configuration of the RVOT and the jet's anterior orientation, procedural techniques for guidewire advancement and device positioning differ significantly from those used in pmVSD closure.

### Type 3: pmVSD with Gerbode shunt

LV to RA shunting caused by a true Gerbode defect due to absence of the atrioventricular component of the membranous septum, which is superior-posterior to the interventricular membranous defect, is exceedingly rare (Figure 1A). More often, LV to RA shunting — “Gerbode shunt” — is due to a pmVSD co-existing with a defect or a cleft in the overlying tricuspid leaflet, or its aneurysmal transformation (Figures 2C1 and 2C2, red arrows).^23^, ^24^, ^25^ This can produce complex shunting not only LV to RA but also LV to RV. The anatomy of this type is more complex with multiple shunts at, below, and above the TV. Device closure has been performed successfully in some centers with some kinds of devices (Nit-Occlud Lê VSD, ADO I, ADO II, MFO), but it has a high risk of tricuspid regurgitation (TR) or stenosis related to the device.^26^, ^27^, ^28^, ^29^

### Type 4: pmVSD with trabecular extension

The defect in type 4 extends inferior-anteriorly. Importantly, this type must be differentiated from “high muscular VSD” in which all borders are muscular and further from the atrioventricular conduction system. This pmVSD is suitable for device closure with many kinds of devices. However, using a device with a stiff disc on the left side may risk left bundle branch block.^5^^,^^30^

### Type 5: pmVSD with inlet extension

**Figure 4:**
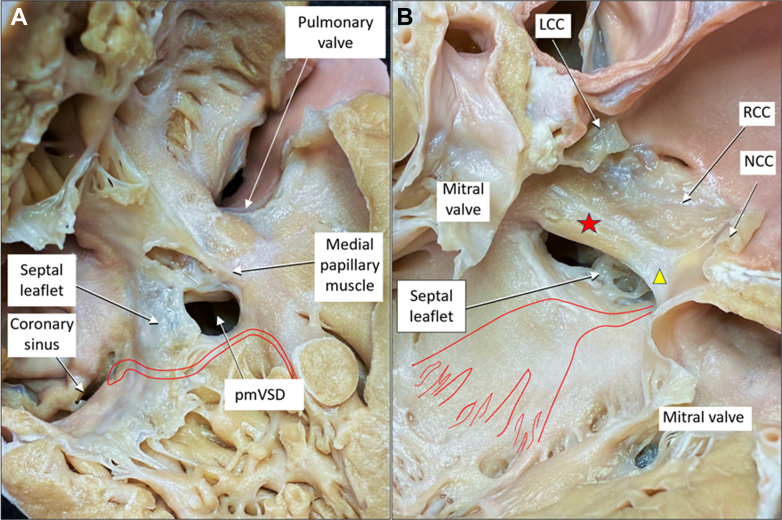
pmVSD With Inlet Extension and the Proximality of the Conduction System (A) The RV aspect shows the pmVSD in relation to the medial papillary muscle located anteriorly and cephalad. The septal leaflet of the tricuspid valve partially covers the defect without other aneurysmal tissue. (B) The LV aspect shows that the posterior border of the defect is formed by mitral–tricuspid fibrous tissue through an extensive remnant of the membranous septum (yellow triangle). Fibrous continuity between mitral–tricuspid and aortic–mitral valves is preserved. However, the defect is separated from the aortic valve by a muscular rim at the antero-cephalad border beneath the right and left coronary cusps (red star). The red lines indicate the conduction tissues close to the inferoposterior defect border. Abbreviations as in Figure 1, Figure 2, Figure 3.

Viewed from the RV, this pmVSD extends inferior-posteriorly beneath the septal leaflet of the TV. At the posterior and inferior borders there is a broad area of fibrous continuity between the mitral, tricuspid, and aortic valves, representing the antero-superior and posterior margins of the defect. When viewed in echocardiography 4-chamber plane, the fibrous continuity between tricuspid and mitral valves appears to roof the VSD. Importantly, these defects are not directly adjacent to the aortic valve but are often covered by TV tissues. The medial papillary muscle is often anterior and superior to these defects. This type shares characteristics with types C and D of the echocardiographic classification. The atrioventricular conduction bundle lies in proximity, coursing along the fibrous posteroinferior rim, making this area particularly sensitive during device-based interventions (Figure 4). The bundle continues for a distance as the common atrioventricular bundle traversing antero-superiorly along the septal crest, or 1 to 2 mm off the crest to the left side of the septum. It then continues as the branching bundle sending fascicles of the left bundle branch that pass beneath the endocardium toward the apical portion of the LV. From the branching bundle, the right bundle branch tends to course through the septal musculature before emerging in the subendocardium on the septomarginal trabeculation, distant from the defect rim. Thus, location wise, the atrioventricular bundle and left bundle branch are vulnerable in some cases.

The development of left bundle branch block following device closure is most often associated with oversized devices, device malalignment to the rest of interventricular septum, or mechanical impingement of the inferoposterior device edge against the left-sided septal rim of the defect. Inflammatory responses around the device may further contribute to the conduction system injury.^31^^,^^32^

**Figure 5:**
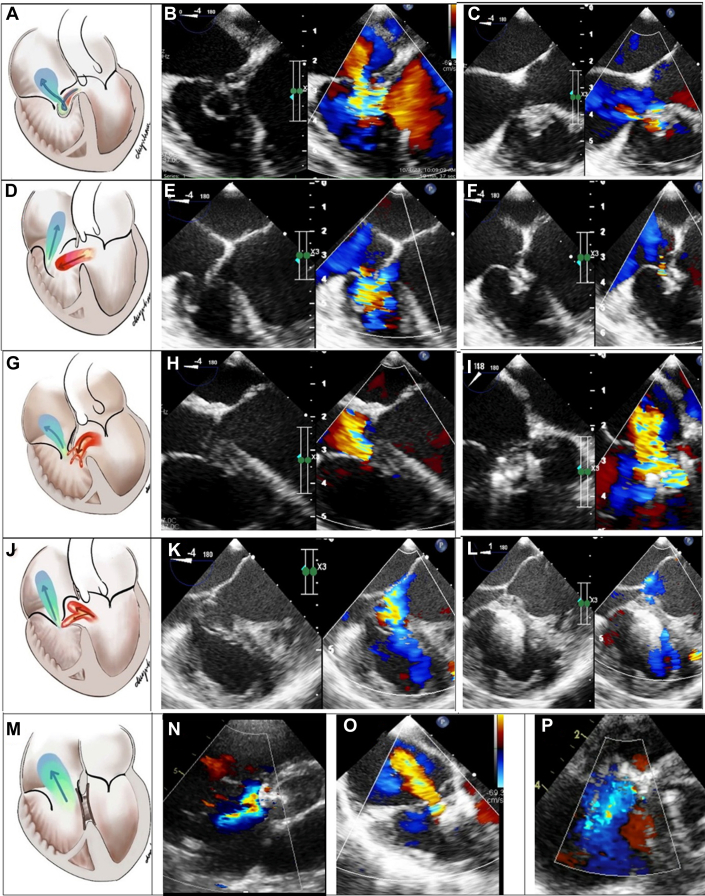
Potential Mechanisms of TR in pmVSD (A, B) Tricuspid regurgitation (TR) due to an LV aneurysm–RA shunt. (C) TR improving after VSD device closure. (D, E) A high-velocity jet through the VSD pushing the anterior leaflet, making poor coaptation. (F) TR improving by stopping the shunt. (G, H) The septal leaflet adhered to the septal aneurysm, causing poor coaptation. (I) TR worsens due to the septal leaflet being trapped between 2 discs of the device. (J, K) Th septal leaflet being pushed by the shunt via inlet-extension pmVSD, causing poor coaptation. (L) TR improving by stopping the shunt with device closure. (M) Device-induced TR due to (N) impingement of the right retention disc of Amplatzer duct occluder II on the TV septal leaflet in a retrograde approach, or (O) entrapment caused by the “clamping force” of 2 retention discs from a LifeTech multifunctional occluder device, or (P) long shank of device protruded into RV. Abbreviations as in Figure 1, Figure 2, Figure 3.

An important issue in device closure for this type of pmVSD is TV involvement and TR. TR in pmVSD may occur in pre-procedural, intra-procedural, and post-procedural periods and visualized on echocardiography (Figure 5). Pre-procedural TR may involve one or more mechanisms related to VSD shunt dynamics, septal leaflet adhesion, and the intrinsic abnormalities of TV (such as cleft of septal leaflet or chordal abnormalities).^33^^,^^34^ When TR is related to VSD shunt dynamics, device closure of the VSD can stop the shunt, restoring free movement and normal coaptation of the anterior or septal leaflet, improving or even eliminating TR (Figures 5A to 5F). When associated with membranous pouches, adhesion of the septal leaflet onto the pouch restricts its movement (Figures 5G to 5I), further compromising valvar function and making TR resolution unpredictable, as device closure may either improve or worsen the TR.

Additionally, when inlet extension is severe, these large defects may not be suitable for device closure because of high risk of device embolization, heart block, and entanglement on TV. Device closure can be done in selected cases with: 1) well-formed septal aneurysm; 2) an opening exit into the RV that is small and far away from the TV annulus; and 3) a trivial to mild TR. A retrograde approach performed through arterial access, typically via the femoral artery, will be suitable for controlling the residual shunt and stability of the device.

### Type 6: confluent type pmVSD

Confluent type is defined when pmVSD extensions are toward more than 1 of 3 regions of the muscular septum. The defect is usually large viewed from the LV side and can extend in two or more directions. Device closure can be achieved in presence of membranous pouch or tags that create multiple holes with different complex shapes. From echocardiographic perspective, the confluent type shares the characteristic of both types A and C or type D due to its broad extensions and its proximities to both aortic and tricuspid valves and aneurysmal tissue. It may need 2 or more devices to completely occlude the hole.

### Type 7: complex type

**Figure 6:**
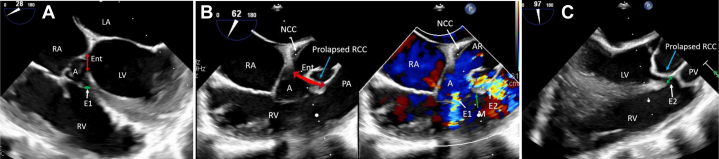
TEE Views of a Complex pmVSD Type (A) Four-chamber view shows a large left-sided entrance (Ent, red double-headed arrow) and a small posteroinferior exit (E1, green double-headed arrow) associated with inlet extension and septal aneurysm (A). (B) Modified short-axis view shows the entrance extending beneath the NCC and the RCC, with RCC prolapse partially covering the defect (blue arrow). On the RV side, the defect divides into 2 exit points: E1 located posteroinferiorly associated with aneurysm (A), and E2 located antero-superiorly just beneath the pulmonary valve (PV) and the PA. Muscular separation (M, green arrow) is visible between the 2 exit points. (C) Modified long-axis view shows the anterosuperior exit point (E2: green double-headed arrow) more clearly. This case is a combination of echocardiographic types A and D and was successfully closed by 2 devices (MFO for E1, fixed inside the aneurysm, ADO II for E2). LA = left atrium; other abbreviations as in Figure 1, Figure 2, and 5.

In complex cases, the pmVSD extends more antero-superiorly resulting in a defect that involves both the membranous septum and the outlet septum. Juxta-arterial and doubly committed VSD, also described as supracristal, are more prevalent in Asian regions, accounting for approximately one-third of VSDs.^35^, ^36^, ^37^, ^38^ In such defects, the superior margin is bordered not by muscle but by a fibrous ridge or by direct fibrous continuity between the pulmonary and aortic valves. When the defect is also associated with a fibrous posteroinferior border, this particular anatomical arrangement is the complex type of pmVSD or, in other words, the pmVSD and juxta-arterial and doubly committed VSD are confluent. The length of the superior fibrous margin may be more or less depending on the extent of the remaining muscular outlet septum posteriorly when viewed from the RV aspect, which provides a muscular rim for device deployment. The smaller the remaining muscular rim of the outlet septum, the larger the doubly committed VSD component, resulting in an increased risk of semilunar valve impingement and device embolism. On the LV aspect, the defect typically presents as a large and often crescent-shaped entrance that hugs the aortic valve. It lies immediately inferior to the interleaflet triangle of RCC and NCC and extends along the entire hingeline of the RCC, increasing the risk of aortic leaflet or sinus distortion and regurgitation. These defects comprise of the differing spatial planes between the remnants of the membranous septum and the anterior portion of any remnant outlet septum. Multiple exit points can sometimes be identified, particularly in large defects, with the muscular separation between them on the RV side; this feature facilitates the possibility of transcatheter device closure. These exit points may be widely spaced, with openings located on distinct planes of the septum or at separate anatomical levels — such as one located posteroinferiorly close to the septal leaflet of TV and one located antero-superiorly close to PV (Figure 6). Because of this complex morphology, this type does not fit neatly into a single echocardiographic classification and often displays overlapping features of multiple defect types, making accurate diagnosis and planning more challenging. When this occurs, closure using a single device may be inadequate to achieve complete occlusion. The deployment of multiple occlusion devices may be necessary to seal all shunt pathways effectively. Reportedly, periventricular device occlusion can be achieved successfully with good outcomes.^39^

### Aneurysmal tissues and their procedural impact

**Figure 7:**
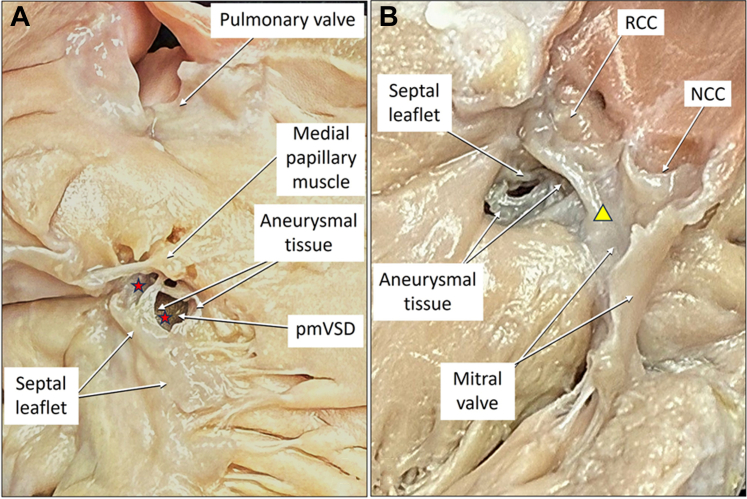
Morphology of pmVSD and Variants Depending on Its ExtensionsAn Inlet pmVSD Partially Covered by Aneurysm (A) RV aspect with multiple exit points of the pmVSD. The aneurysmal tissue is separate from the septal leaflet and chordae. The gap (red stars) between the aneurysmal tissue and the septal leaflet will facilitate the device closure with lower risk of tricuspid valve entrapment. (B) LV aspect with a large entrance of the pmVSD and a remnant of membranous septum (yellow triangle), which is suspended beneath the aortic valve, serving as a superior rim of the defect. Abbreviations as in Figures 1 and 2.

**Figure 8:**
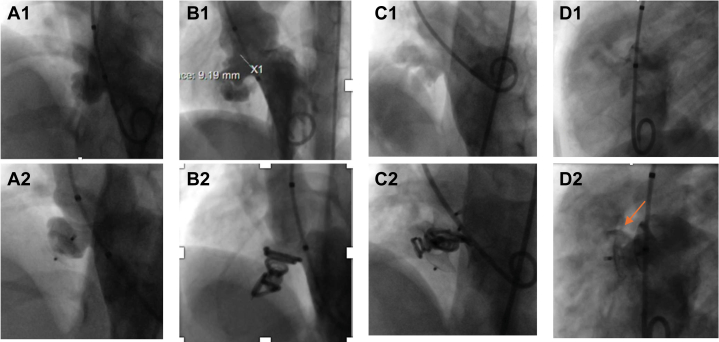
Various pmVSD Configurations With Septal Aneurysms (A1) A pmVSD with a single exit was closed via a retrograde approach using a 6/4 mm ADO II (A2), ensuring the left disc lay completely within the aneurysm to avoid tricuspid regurgitation and protect the aortic valve. (B1) A pmVSD and septal aneurysm presenting multiple RV exits was completely occluded from the LV side with a 14/8 mm Nit-Occlud Lê VSD (B2). (C1) A pmVSD with widely separated RV exits was managed with a combination of one 14/8 mm Nit-Occlud Lê VSD and two 6/4 mm ADO II devices (C2). (D1) A pmVSD featuring 2 distinct orifices was addressed using a 6-mm Occlutech pmVSD device, leaving a small residual shunt (arrow) intentionally unclosed (D2). Abbreviations as in Figures 1 and 5.

Additional to all these anatomical types, consideration must be given to the presence of any aneurysmal tissue in the vicinity as described in type 5. In many cases, the RV aspect of the defect may be partially occluded by surrounding structures appearing such as an aneurysm on imaging. This aneurysm, sometimes perceived as an aneurysm of the membranous septum, mostly comprises abnormal tissues and folds of the tricuspid leaflet and chords attempting to close the defect or accessory tissue tags or pouches from the tricuspid or aortic leaflets, giving single or multiple holes in the aneurysmal tissue (Figure 7).^40^, ^41^, ^42^, ^43^, ^44^ In the context of transcatheter closure of pmVSDs, aneurysmal tissue often provides a valuable landing zone for device anchoring, serving to stabilize the occluder while minimizing interference with nearby critical structures, such as the aortic valve and the conduction system (Figures 7 and 8).

In certain anatomical variants, the aneurysm is a key enabler of successful intervention.

In large pmVSDs with inlet extension, an aneurysm may function as an additional septal rim, facilitating device retention and offering a protective buffer for the conduction tissue along the posteroinferior border. In such anatomies, the risk of complications such as heart block or TR is typically reduced. Conversely, in non-aneurysmal VSDs, closure becomes more challenging due to greater exposure of the conduction system.

In echocardiographic type A pmVSDs (without an aortic rim), true overriding of the aortic valve, by deploying the device within the aneurysmal cavity, can shield the aortic leaflets from valve impingement and mitigate the risk of aortic regurgitation.

An important anatomical feature to evaluate during preprocedural planning is whether the septal leaflet is involved in the aneurysm. In the echocardiographic classification, pmVSDs with aneurysmal tissue related to septal leaflet of TV are classified as type D, whereas those formed by fibrous tissue ingrowth and accessory tags without any adhesion of the septal leaflet are type C. A distinguishing feature of echocardiography type C is the presence of a gap between the aneurysm and the septal leaflet and its chordae, providing a safe landing zone for the right disc of the occluder (Figure 7). In these cases, the device can be deployed without impinging upon the septal leaflet, thus avoiding worsening TR. In contrast, when the septal leaflet is adhered to the aneurysm, its mobility is restricted, and deployment of the right disc may result in entrapment of the leaflet, leading to worsening TR.

In cases where the aneurysm is floppy or mobile, the width of the jet flow across the exit points on color Doppler imaging is typically broad, reflecting an unstable and poorly supported structure. In such scenarios, an antegrade approach with a pulling maneuver during the procedure is advisable to test the mechanical integrity and stability of the aneurysmal pouch before deploying the occlusion device.

## Conclusions

pmVSDs exhibit a wide spectrum of anatomical variations, each carrying distinct implications for transcatheter closure. Although the echocardiographic classification is helpful, there is a wide range of anatomical variants within each type. Precise anatomical assessment, particularly of outlet and inlet extensions, valve proximity, aneurysmal tissue, and the course of the conduction system, is essential for selecting appropriate candidates and minimizing procedural risks. Special considerations, such as aortic valve prolapse or overriding anatomy, demand tailored interventional strategies and meticulous device positioning. Detailed and careful imaging modalities to accurately characterize the location, extent, and surrounding anatomy of the defect remains fundamental to ensuring safe and effective intervention.

## Funding Support and Author Disclosures

All authors have reported that they have no relationships relevant to the contents of this paper to disclose.
